# Scientific investigation of crude alkaloids from medicinal plants for the management of pain

**DOI:** 10.1186/s12906-016-1157-2

**Published:** 2016-06-13

**Authors:** Mohammad Shoaib, Syed Wadood Ali Shah, Niaz Ali, Ismail Shah, Shafi Ullah, Mehreen Ghias, Muhammad Nawaz Tahir, Farah Gul, Sohail Akhtar, Abd Ullah, Wajid Akbar, Asad Ullah

**Affiliations:** Department of Pharmacy, University of Malakand, Malakand, Khyber Pakhtunkhwa Pakistan; Department of Pharmacology, Institute of Basic Medical Sciences, Khyber Medical University, Peshawar, Khyber Pakhtunkhwa Pakistan; Department of Physics, University of Sargodha, Punjab, Pakistan; Pharmacology Section, PCSIR Laboratories Complex, Peshawar, Khyber Pakhtunkhwa Pakistan; Department of Statistics, University of Malakand, Malakand, Khyber Pakhtunkhwa Pakistan

**Keywords:** Crude alkaloids, *Woodfordia fruticosa*, *Peganum harmala*, Traditional uses, Pain, Naloxone, Opioid

## Abstract

**Background:**

Tissue damage is associated with pain, which is an alarming sign. Aspirin and morphine have been widely used in recent decades for management of pain. Medicinal herbs have been in use for treatment of different diseases for centuries. Many of these herbs possess analgesic activity with relatively less incidences of adverse effects. The strong positive correlation of alkaloids in medicinal plants for analgesic activity persuades an intention to determine possible analgesic activity of total alkaloids extracted from the selected medicinal plants using animal models to answer its possible mechanisms.

**Methods:**

Crude alkaloids from selected medicinal plants (*Woodfordia fruticosa, Adhatoda vasica, Chenopodium ambrosioides, Vitex negundo, Peganum harmala* and *Broussonetia papyrifera*) were extracted as per reported literature. The test crude alkaloids were screened foracute toxicity study. Writhings induced by acetic acid, tail immersion method and formalin-induced nociception assay procedures were used for possible analgesic effects of the crude alkaloids.

**Results:**

Crude alkaloids were safe up to dose of 1250 mg/kg body weight in mice. The alkaloids significantly reduced the abdominal constrictions, and increased the time for paw licking response in both phases with a significant raise in latency time in nociception models (*P* ≤ 0.05). Moreover, the antinociceptive response was significantly attenuated by pretreatment with naloxone suggesting involvement of the opioid receptors for possible antinociceptive action.

**Conclusions:**

Crude alkaloids of *Woodfordia fruticosa* and *Peganum harmala* showed prominent analgesic potentials through inhibition of peripheral as well as central nervous system mechanisms. Further work is required for isolation of the pharmacologically active constituents.

## Background

International Association for the Study of Pain (IASP) defines pain as “a disagreeable sensory and arousing experience coupled by real or potential tissue damage” [1]. Usually pain is thought to be an alarming sign of actual or apparent tissue damagethat warns the subject for possible unwanted outcome. Therefore,the subject seeks averting reaction for possible defense [2]. Aspirin and morphine are in use for analgesic purposes in recent decades. Opioids and nonsteroidal anti-inflammatory drugs (NSAIDs) have been reported to relieve pain sometimes by 50 % in 30 % of recipients [3]. The uses of these analgesics are associated with incidences of adverse effects like opiates cause physical dependence, tolerance and addiction. NSAIDs are frequently associated with gastrointestinal disorders like gastric or duodenal ulceration [4].

This necessitates for discovery of relatively safe alternatives for treatment of pain. Medicinal herbs have been used for therapeutic purposesfor centuries. Many of these herbs had been used for pain management without any evident adverse effects [5]. Ethno-pharmacologically guided research has brought considerable contributions to new drug development [6, 7]. There has been an increasing interest to find new and safe anti-inflammatory and analgesic drugs from natural sources including medicinal plants [8]. Medicinal plants have been very useful source for lead structure for subsequent synthetic modification and optimization of bioactivity.

Alkaloids are naturally occurring active diverse group of secondary metabolites in plants that has been used in medicine for hundreds years [9].

Plants like *Woodfordia fruticosa, Adhatoda vasica*, *Chenopodium ambrosioides, Viburnum cotinifolium, Vitex negundo*, *Peganum harmala* and *Broussonetia papyrifera *have been investigated scientifically for presence of alkaloids with reference to their ethno pharmacological profile in pain management [10, 11–16].

Analgesic activity of alkaloids isolated from plants is reported with different mechanistic approaches [17–20]. The strong positive correlation of alkaloids in medicinal plants for analgesic activity persuades an intention to determine possible analgesic activity of total alkaloids extracted from the mentioned medicinal plants using animals’ model.

## Methods

### Plant materials

The collected plant species namely *Woodfordia fruticosa* (voucher specimen Wf-01-2015, *Lythraceae,* Swat, *aerial parts), Adhatoda vasica syn. Justicia adhatoda* (voucher specimen Av-02-2015, Acanthaceae, Kohat, leaves), *Chenopodium ambrosioides* (voucher specimen Ca-03-2015, *Chenopodiaceae, Peshawar, leaves), Vitex negundo* (voucher specimen Vn-04-2015, Verbenaceae, Swat, *aerial parts*), *Peganum harmala* (voucher specimen Ph-05-2015, *Zygophyllaceae*, Peshawar, *aerial parts*) and *Broussonetia papyrifera* syn. *Morus papyrifera* (voucher specimen Bp-06-2015, Moraceae, Dir Lower, *aerial parts*) were identified by Dr. Jehandar Shah, ex Vice Chancellor, University of Malakand. The materials were dried under shade. Respective voucher specimens were submitted to the herbarium of Department of Botany, University of Malakand, Dir Lower, Khyber Pakhtunkhwa.

## Extraction of crude alkaloids

The crude alkaloidal extracts from the plants were obtained according to the method of Harborne with slight modifications. Briefly, dried powdered plants materials (100 g) were defatted using Soxhlet apparatus (Quickfit, UK) followed by an extraction process with 10 % acetic acid (200 ml) in ethanol for 24 h in Soxhlet apparatus. The extract was concentrated using rotary evaporator till a concentrated mass. The pH was adjusted to 9 by the addition of concentrated ammonium hydroxide solution. It was then extracted with chloroform (50 ml) three times. During extraction, the contents were subjected to vigorous shaking. The chloroform layer was separated using separating funnel. The chloroform portion was concentrated using rotary evaporator. Presence of alkaloids was confirmed with Dragendorff’s, Mayer’s or picric acid reagent. The alkaloids were stored in a clean amber glass vial at 4 °C. Similar procedure was used to extract crude alkaloids from the aforementioned specimens. The alkaloids were successively labeled as *Woodfordia fruticosa* crude alkaloid (Wf Cr.A)*, Adhatoda vasica *crude alkaloid (Av Cr.A),* Chenopodium ambrosioides *crude alkaloid (Ca Cr.A)*, Vitex negundo *crude alkaloid (Vn Cr.A),* Peganum harmala *crude alkaloid (Ph Cr.A) and *Broussonetia papyrifera *crude alkaloid (Bp Cr.A) [21–23].

## Drugs and standards

Analytical grade chemicals were used in the experiments. Acetylcholine was purchased from BDH, Poole, England. Other chemicals were of E. Merck grade. All the stock solutions were prepared in distilled water. Fresh dilutions were made in normal saline.

## Animals

Either sex Balb/C mice (weight range: 25-30 g) were purchased from National Institute of Health Islamabad. They were housed in the animal house of the University of Malakand, Pakistan under a controlled environment (23-25 °C). The animals were fasted overnight before the start of the experiments. They were treated as per the principles mentioned in the “Animals Byelaws 2008 of University of Malakand (Scientific Procedures Issue-I)”. Ethical Committee of the Department of Pharmacy, constituted under the approved Animals Byelaws 2008 of University of Malakand, endorsed the study protocols.

## Acute toxicity

Acute toxicity study for crude alkaloids was carried out as per procedure reported by Lorke [24]. Male mice were selected by random sampling technique and three groups for individual test sample for two phases were used for the determination of LD_50_. In the initial phase, the mice were orally treated with 125, 250 and 500 mg/kg of the alkaloidal extract. All test samples were tested in similar manner. The groups were observed for possible mortality in 24 h. In the second phase, the mice were treated with the test alkaloids extract in doses of 750, 1000 and 1250 mg/kg. Death toll was noted in 24 h. LD_50_was calculated [24, 25].

## Acetic acid-induced writhing

Analgesic activity was carried out in mice. Writhings were induced using acetic acid. Briefly, Balb/ C male mice were divided into different groups having six animals in each group. The animals were pretreated with crude alkaloids of various medicinal plants at a dose of 50 mg/kg (i.p.). Diclofenac sodium (50 mg/kg, i.p.) was used as standard analgesic agent. The test samples and standard drugs were administered 1 h before of intraperitoneal injection of 1 % (v/v) acetic acid (0.1 ml/10 g). Five minutes after the injection (i.p) of acetic acid, counted the number of writhing following 10 min. Negative control group received carboxy methyl cellulose (CMC) 0.5 % and vehicle only [26].

### Formalin test

This test was performed by the method of assessing the licking response of formalin-induced edema in paw of mice. 20 μl of 1 % formalin prepared in 0.9 % saline was administered (s.c) to the dorsal hind paw and immediately placed in the transparent box for observing the licking response. The duration of reaction time (paw licking or biting) was determined between 0 and 5 min (first phase) and 15–30 min (second phase). Animals in different groups were treated test samples (50 mg/kg, i.p), indomethacin (10 mg/kg, i.p) and morphine (5 mg/kg, i.p), 30 min prior to administration of formalin. Naloxone (2 mg/kg, i.p) was administered 20 min prior to treatment of animals with test samples and standards. Control animals received the vehicle (0.1 ml/10 g). The reaction time of the animals in respective groups was compared to control group and expressed as percent inhibition [27].

## Tail immersion method

The animals were divided into groups of six animals each. To prelabelled group (CMC 0.5 %), morphine (5 mg/kg), and crude alkaloids at a dose of 50 mg/kg of different medicinal plants were given. Time for tail withdrawal from the water was measured before and after drug treatment in a regular interval of 15, 30, 45, 60, 75 and 90 min by immersing the tail tips (1-2 cm) of the mice in water bath thermostatically maintained at temperature of 55 ± 1 °C with a cut-off time of immersion at 10 s. The actual flick response of mice was measured by stop watch and results were compared with control and standard group [27].

## Statistical analysis

Data are presented as mean ± SEM. Analysis of variance and Dunnett’s test is statistically manipulated with GraphPad prism software version 5.01.

## Results

After 24 h of observation, in acute toxicity test, no behavioral and physical changes in mice were observed at doses below 1250 mg/kgbody weight for crude alkaloids.

**Fig. 1:**
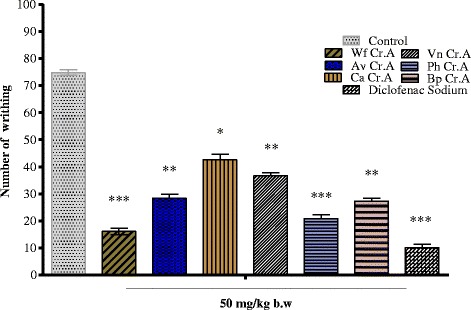
Analgesic activity of crude alkaloids using acetic acid induced writhing model. All the values are expressed as mean ± SEM (*n* = 6). ^*^ *P* < 0.05, ^**^ *P* < 0.01, ^***^ *P* < 0.001 when compared to control group

The effect of crude alkaloids in the acetic acid induced writhing model is mentioned in Fig. 1. The compounds antagonized abdominal constrictions at a dose of 50 mg/kg i.p., maximum pain alleviation (78.45 %, *P* < 0.001, *n* = 6) was observed for *Woodfordia fruticosa* crude alkaloid followed by *Peganum harmala* crude alkaloid (72.13 %, *P* < 0.001, *n* = 6). Other alkaloids also produce protection against acetic acid to 63.50, 61.99, 51.10 and 43.03 respectively for *Broussonetia papyrifera* crude alkaloid, *Adhatoda vasica* crude alkaloid, *Vitex negundo* crude alkaloid and *Chenopodium ambrosioides* crude alkaloid. The standard diclofenac sodium produce significant protection of 86.42 %, *P* < 0.001, *n* = 6 at a dose of 50 mg/kg i.p.

**Fig. 2:**
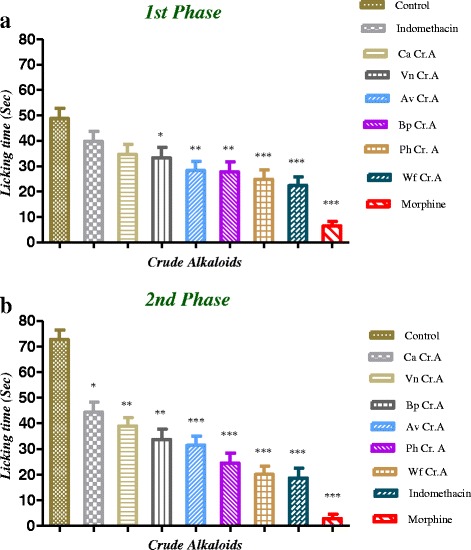
**a** Analgesic effect of extracted crude alkaloids on formalin induced licking response in first phase of the test. Values are mean ± SEM, *n* = 6, ^*^ *P* < 0.05, ^**^ *P* < 0.01, ^***^ *P* < 0.001 significantly different compared with control. **b**: Analgesic effect of extracted crude alkaloids on formalin induced licking response in second phase of the test. Values are mean ± SEM, *n* = 6, ^*^ *P* < 0.05, ^**^ *P* < 0.01, ^***^ *P* < 0.001 significantly different compared with control

The results of the crude alkaloids in post formalin induced flinching behavior are presented in Table 1 and Fig. 2. The compounds produced significant reduction in noxious stimulation in both phases. Pretreatment of test samples at the dose of 50 mg/kg i.p. showed marked attenuation of noxious stimulation in both phases in which *Woodfordia fruticosa* crude alkaloid (Wf Cr.A) was dominant with 72.37 and 54.22 % in the both phases, respectively. Similar tendency was produced by *Peganum harmala* crude alkaloid (Ph Cr.A) with 66.31 and 49.16 % control in the 1st and 2nd phases respectively (Fig. 2a and b). The other crude alkaloids also produced moderate to good reduction in noxious stimulation in both phases. The standard indomethacin at a dose of 10 mg/kg produced significant effects of 74.37 % in second phase while less effects were produced in first phase. The centrally acting standard morphine at a dose of 5 mg/kg significantly reduced the noxious stimulation in both phases upto 96.11 % (*P* < 0.001, *n* = 6) and 86.86 % (*P* < 0.001, *n* = 6) respectively.

**Table 1:** Effects of crude alkaloids and standardson formalin-induced paw-licking response in mice

Treatment/Dose	Licking time (Sec)		Inhibition (%)	
	1st Phase	2nd Phase	1st Phase	2nd Phase
Control (2 % Tween 80)	48.83 ± 1.627	72.83 ± 1.287	----	----
Wf Cr.A 50 mg	22.35 ± 1.231^***^	20.12 ± 1.163^***^	54.22	72.37
Av Cr.A 50 mg	28.31 ± 1.347^**^	31.45 ± 1.342^***^	42.14	56.81
Ca Cr.A 50 mg	34.75 ± 1.441^*^	44.39 ± 1.568^*^	28.68	39.04
Vn Cr.A 50 mg	33.35 ± 1.604^*^	38.93 ± 1.195^**^	31.82	46.54
Ph Cr.A 50 mg	24.85 ± 1.289^***^	24.53 ± 1.391^***^	49.16	66.31
Bp Cr.A 50 mg	27.80 ± 1.468^**^	33.79 ± 1.416^**^	43.09	53.60
Indomethacin (10 mg)	39.83 ± 1.541	18.66 ± 1.542^***^	18.43	74.37
Morphine (5 mg)	6.416 ± 1.165^***^	2.83 ± 1.260^***^	86.86	96.11

All the values are expressed as mean ± SEM. ^*^ *P* < 0.05, ^**^ *P* < 0.01 and ^***^ *P* < 0.001 when compared to control group (one way ANOVA followed by Dunnetts: compare all vs control test)

In thermal nociception model, the tail immersion test has been carried out for finding the central effect of crude alkaloids. In this test, *Woodfordia fruticosa* crude alkaloid at 50 mg/kg (i.p) showed its highest analgesic response at all time points (15-90 min) in comparison with alkaloids of other plants. The results of these crude alkaloids are shown in Table 2. Morphine was used as reference at a dose of 5 mg/kg, i.p.

**Table 2:** Analgesic effects of crude alkaloids and standard drugs using tail flick method

	Time in Sec (Tail Flick)/Response (%)					
Treatment/Dose	15 min	30 min	45 min	60 min	75 min	90 min
Control (2 % Tween 80)	0.78 ± 1.30	0.88 ± 1.21	0.98 ± 1.021	0.93 ± 1.381	0.86 ± 1.025	0.92 ± 1.401
Wf Cr.A 50 mg	1.01 ± 1.12* (22.70 %)	1.38 ± 1.112* (36.23 %)	1.68 ± 1.131** (41.66 %)	2.13 ± 1.124** (56.33 %)	2.41 ± 1.163*** (64.31 %)	2.15 ± 1.138*** (57.20 %)
Av Cr.A 50 mg	0.92 ± 1.20 (15.21 %)	1.14 ± 1.411* (22.52 %)	1.41 ± 1.161** (30.49 %)	1.48 ± 1.201** (37.16 %)	1.65 ± 1.206*** (47.87 %)	1.57 ± 1.282*** (41.40 %)
Ca Cr.A 50 mg	0.89 ± 1.30 (12.35 %)	1.09 ± 1.411* (19.85 %)	1.35 ± 1.161** (27.40 %)	1.39 ± 1.231** (33.09 %)	1.53 ± 1.226*** (43.79 %)	1.49 ± 1.218*** (38.25 %)
Vn Cr.A 50 mg	0.90 ± 1.10 (13.81 %)	1.12 ± 1.521* (21.75 %)	1.38 ± 1.611** (28.98 %)	1.40 ± 1.421** (33.80 %)	1.55 ± 1.261*** (44.69 %)	1.51 ± 1.618*** (39.27 %)
Ph Cr.A 50 mg	0.97 ± 0.82 (19.58 %)*	1.23 ± 1.135* (28.45 %)	1.70 ± 1.141** (42.35 %)	2.08 ± 1.511** (55.28 %)	2.33 ± 1.133** (63.09 %)	2.04 ± 1.123** (54.90 %)
Bp Cr.A 50 mg	0.90 ± 0.97 (13.81 %)	1.11 ± 1.411* (20.42 %)	1.37 ± 1.261** (28.46 %)	1.42 ± 1.301** (34.50 %)	1.57 ± 1.237*** (45.22 %)	1.53 ± 1.218*** (39.90 %)
Standard (Morphine 5 mg)	1.54 ± 1.24** (49.35 %)	2.11 ± 1.066** (58.29 %)	4.52 ± 1.038*** (78.31 %)	6.12 ± 1.054*** (84.80 %)	4.98 ± 1.309*** (82.70 %)	4.74 ± 1.074*** (80.59 %)
Wf Cr.A (50 mg) + Naloxone (2 mg)	0.82 ± 0.83	0.81 ± 1.165	0.93 ± 1.049	0.99 ± 1.308	0.87 ± 1.029	0.83 ± 1.461
Av Cr.A (50 mg) + Naloxone (2 mg)	0.96 ± 0.91	0.86 ± 1.036	0.88 ± 1.131	0.94 ± 1.047	0.89 ± 1.381	0.84 ± 1.069
Ca Cr.A (50 mg) + Naloxone (2 mg)	0.84 ± 1.08	0.91 ± 1.044	0.84 ± 1.045	0.89 ± 1.642	0.94 ± 1.263	0.78 ± 1.422
Vn Cr.A (50 mg) + Naloxone (2 mg)	0.94 ± 1.03	0.88 ± 1.051	0.81 ± 1.521	0.83 ± 1.342	0.86 ± 1.061	0.87 ± 1.356
Ph Cr.A (50 mg) + Naloxone (2 mg)	0.81 ± 0.69	0.96 ± 1.031	1.05 ± 1.271	0.97 ± 1.535	0.91 ± 1.251	0.93 ± 1.034
Bp Cr.A 50 mg + Naloxone (2 mg)	0.88 ± 1.124	0.81 ± 0.89	0.94 ± 1.034	0.86 ± 1.324	0.93 ± 1.304	0.94 ± 1.263
Morphine (5 mg) + Naloxone (2 mg)	0.76 ± 0.82	0.87 ± 1.336	0.96 ± 1.204	0.95 ± 1.641	0.89 ± 1.035	0.97 ± 1.432

All the values were expressed as mean ± SEM. ^*^ *P* < 0.05, ^**^ *P* < 0.01 and ^***^ *P* < 0.001 when compared to control group (one way ANOVA followed by Dunnetts: compare all vs control test)

The analgesic response of alkaloids was significantly antagonized by naloxone at all the time intervals. The crude alkaloids extracted from different medicinal plants exhibited good to significant analgesic effect in tail immersion test, indicating the involvement of both spinal and supraspinal analgesic pathways.

Upon the administration of the nonselective opioid receptor antagonist naloxone, the analgesic response of crude alkaloids was antagonized.

## Discussions

Plants have been in continuous use for treatment of various ailments since creature of human beings. Today many of the current drugs are from natural sources [28]. Pain management sometimes requires more than one drug therapy. Thus the practice of polypharmacy carries risks of adverse drug reactions and or side effects. Therefore, search for new drugs with the same therapeutic efficacy with relatively less frequency of side effects is the need of the time [29, 30].

This study helped us in understanding the possible mechanisms of potential analgesic effect of the test alkaloids that work through inhibition of central nervous system and peripheral nervous system. The abdominal constriction induced by acetic acid is thought to be due to the involvement of peripheral mechanisms, while tail immersion test is thought to be due to central mechanisms [31]. Formalin test is used for both peripheral and central mechanisms [32].

Acetic acid-induced writhing is a well-recommended model to assess the analgesic proprieties of drugs having analgesic and anti-inflammatory activity. Acetic acid performs its action by release of endogenous mediators like prostaglandins from arachidonic acid through cyclooxygenase enzymes. These prostaglandins stimulate the nociceptive neurons [33, 34] with induction of pain sensation. The abdominal constriction response may also be due to the activation of local peritoneal receptors [35] and involved prostanoids mediators.

NSAIDs possess analgesic activity by inhibiting the cyclooxygenase pathway and synthesis of the prostaglandins that confirm the involvement of peripheral mechanism in inhibition of pain [36]. Thus NSAIDs in writhing model are used as positive control for inhibition of pain that works through peripheral mechanism [37, 38]. Therefore, the analgesic action of crude alkaloids seems to be due to inhibition of cyclooxygenase or 5-lipoxygense pathways that may be attributed to inhibition of inflammatory cytokines and interleukins [39].

The formalin test model is used to investigate on the ability to exert peripheral and or central analgesic effects as it assays biphasic characteristics, labeled as the early and late phases that occur as a result of formalin administration [40]. The early phase is a neurogenic pain resulting from an acute response towards direct action of formalin on nociceptors within the intraplantar region, while the late phase is considered as an inflammatory-mediated pain resulting from a tonic response due to the release of inflammatory mediators [41]. There may be activation of the neurons in the dorsal horns of the spinal cord as well [42, 43]. Generally, the peripherally-acting drugs like aspirin blocks the synthesis of prostaglandins,which inhibit the late phase only, while narcotic analgesics (centrally-acting) inhibit nociception in both phases of the formalin test [44, 45].

Tail immersion test is selective for morphine like compounds. It tests the reflex responses involved through the spinal cord for possible sensation of nociceptive stimuli [46]. The brain and spinal cord play an important role in central pain mechanism. Dorsal part of spinal cord is rich withsubstance P, endogenous opioids, somatostatin, and other inhibitory hormones which are the targets of pain and inflammation [47]. The results of the tail immersion test indicated that antinociceptive effect of crude alkaloids and standard morphine was reversed by naloxone suggests the involvement of opioid receptors at level of spinal cord. It has been suggested that μ2- and δ- opioid receptors are involved in spinal mechanism [48]. Therefore, it can be anticipated that the central analgesic effects of extracted crude alkaloids may be prominent on μ-opioid receptors.

The crude alkaloidal extractsof different medicinal plants possess analgesic potentials possibly through inhibion of central and peripheral pain mediators. The antinociceptive activity confirms traditional uses of the aforementioned medicinal plants for the management of pain.

## Conclusion

Crude alkaloids of *Woodfordia fruticosa* and *Peganum harmala* showed prominent analgesic potentials that requires further work for isolation of pharmacologically active constituents.

## Abbreviations

Av Cr.A, *Adhatoda vasica* crude alkaloid; Bp Cr.A, *Broussonetia papyrifera* crude alkaloid; Ca Cr.A, *Chenopodium ambrosioides* crude alkaloid; CMC, Carboxy methyl cellulose; Ph Cr.A, *Peganum harmala* crude alkaloid; s.c, subcutaneous; Vn Cr.A, *Vitex negundo* crude alkaloid; Wf Cr.A, *Woodfordia fruticosa* crude alkaloid.
